# Development of lysosome-mimicking vesicles to study the effect of abnormal accumulation of sphingosine on membrane properties

**DOI:** 10.1038/s41598-017-04125-6

**Published:** 2017-06-21

**Authors:** Ana C. Carreira, Rodrigo F. M. de Almeida, Liana C. Silva

**Affiliations:** 1 iMed.ULisboa –Research Institute for Medicines, Faculdade de Farmácia, Universidade de Lisboa, 1649-003 Lisboa, Portugal; 2 Centro de Química e Bioquímica, DQB, Faculdade de Ciências, Universidade de Lisboa, Campo Grande, 1749-016 Lisboa, Portugal; 3 Centro de Química-Física Molecular and Institute of Nanoscience and Nanotechnology, Instituto Superior Técnico, Universidade de Lisboa, Av. Rovisco Pais, 1049-001 Lisboa, Portugal

## Abstract

Synthetic systems are widely used to unveil the molecular mechanisms of complex cellular events. Artificial membranes are key examples of models employed to address lipid-lipid and lipid-protein interactions. In this work, we developed a new synthetic system that more closely resembles the lysosome – the lysosome-mimicking vesicles (LMVs) – displaying stable acid-to-neutral pH gradient across the membrane. To evaluate the advantages of this synthetic system, we assessed the distinct effects of sphingosine (Sph) accumulation in membrane structure and biophysical properties of standard liposomes (no pH gradient) and in LMVs with lipid composition tuned to mimic physiological- or NPC1-like lysosomes. Ternary 1-palmitoyl-2-oleoyl-sn-glycero-3-phosphocholine (POPC)/Sphingomyelin (SM)/Cholesterol (Chol) mixtures with, respectively, low and high Chol/SM levels were prepared. The effect of Sph on membrane permeability and biophysical properties was evaluated by fluorescence spectroscopy, electrophoretic and dynamic light scattering. The results showed that overall Sph has the ability to cause a shift in vesicle surface charge, increase membrane order and promote a rapid increase in membrane permeability. These effects are enhanced in NPC1- LMVs. The results suggest that lysosomal accumulation of these lipids, as observed under pathological conditions, might significantly affect lysosomal membrane structure and integrity, and therefore contribute to the impairment of cell function.

## Introduction

Sphingosine (2*S*,3*R*-d-erythro-2-amino-1,3-octadec-4*E*-ene-diol) is one of the most abundant sphingoid backbone in mammalian sphingolipids (SLs)^1^. It results from the degradation of ceramide (Cer) by ceramidases in different sub-cellular locations^2^. In the lysosome, Sph is generated through the hydrolysis of Cer by acid ceramidase. The amino group of Sph is protonated under acidic conditions and therefore it needs a transporter (currently unknown) to facilitate its egress from the acidic compartments^3^. Free Sph levels are generally maintained low, due to its rapid further metabolization into important signaling molecules. This lipid can be converted into sphingosine-1-phosphate through the phosphorylation of C1 hydroxyl group or acylated through the action of different ceramide synthases to produce Cer^2, 4, 5^.

Sph is itself a bioactive lipid and it plays an active role in different biological processes, such as proliferation^6^, and apoptosis^7, 8^. Moreover, Sph has been implicated in the regulation of the activity of various enzymes, including protein kinases^7, 9–13^. Most of these enzymes do not have a *bona fide* Sph-binding site identified. They are amphitropic proteins, a feature that is important for their activity and regulation^14^. This suggests that the regulatory effect of Sph can in part be exerted at the membrane level, namely through physical changes that might affect the distribution of lipids and proteins and consequently trigger different cellular events.

As an important bioactive molecule, Sph has its cellular levels tightly regulated^15^. However, in some situations, like in Niemann Pick Disease type C1 (NPC1), Sph abnormally accumulates in the late endosomes and lysosomes of cells, inducing the secondary accumulation of Chol, SM and other SLs^16^. These lipid changes might have important consequences at the level of membrane structure, organization and properties, which might influence lysosomal-associated events^3, 17^ or even compromise lysosomal integrity^18, 19^.

**Figure 1:**
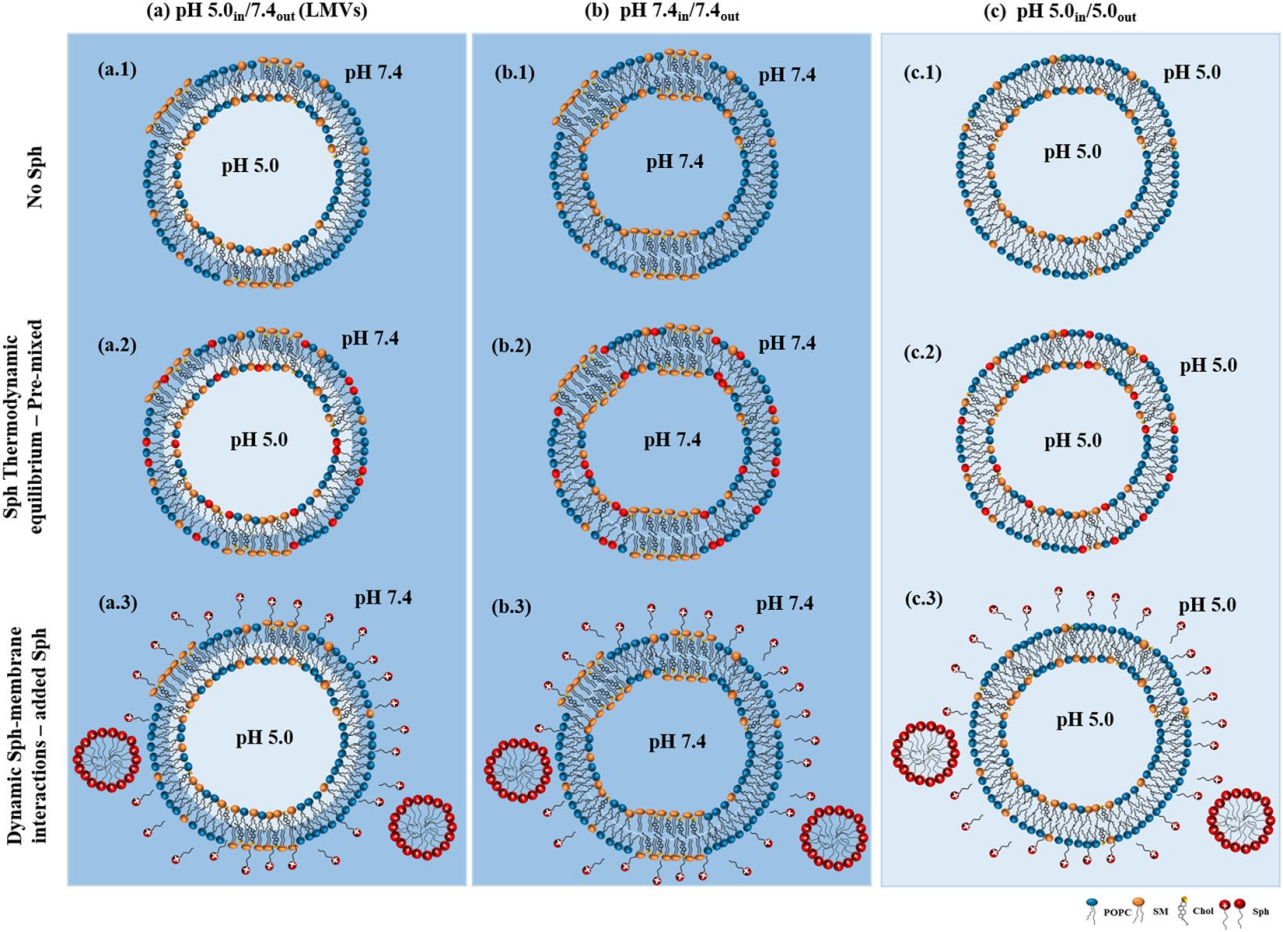
Schematic representation of the synthetic systems used in this study. Artificial membrane systems composed by POPC/SM/Chol were prepared at different pH conditions. (**a**) A new synthetic system that more closely resembles the lysosome – the lysosome-mimicking vesicles (LMVs), displaying internal acidic pH and external neutral pH. (pH 5.0_in_/7.4_out_) was prepared through buffer exchange by gel filtration and compared with data from simpler systems prepared at (**b**) pH 7.4_in_/7.4_out_ and (**c**) pH 5.0_in_/5.0_out_. To address the effects of Sph in the membrane, studies were performed in equilibrated sample conditions (**a.2, b.2, c.2**) (i.e., Sph pre-incorporated in the membrane prior to vesicle preparation) and (**a.3, b.3, c.3**) upon dynamic interaction with the membrane (i.e., Sph externally added to pre-formed POPC/SM/Chol vesicles). In addition, vesicles with different lipid compositions were prepared in order to evaluate the effect of Sph on the properties of membranes containing low (physiological-like) or high (NPC1-like) levels of Chol and SLs. The higher the levels of Chol and SM of the mixtures the higher the *l*_o_ phase fraction (X*l*_o_) (see methods section and Supplementary Table S1 for further details).

In recent years, efforts have been made in order to understand the impact of Sph in membrane organization. It was demonstrated that Sph has the ability to change the membrane physical organization, promoting the formation of more ordered domains^18, 20–26^. The order of these domains depends not only on membrane lipid composition but also on the surrounding pH environment^21, 26, 27^. It is likely that Sph-induced alterations in membrane properties are closely related to the physico-chemical changes experienced by this lipid. The protonation state^23, 28^ and the H-bonding network of Sph^29^ are susceptible to pH changes occurring within the range of different physiological pH environments, suggesting that Sph might have a different behavior depending on its sub-cellular location. For instance, the fact that Sph might become more positively charged in the acidic conditions of the lysosome may strongly affect its interaction with the surrounding lipids and interfere with the formation and maintenance of Sph domains. It was recently shown that by interacting with negatively charged lipids, commonly found in biological membranes, including lysosomal membranes, Sph contributes to the formation of transient non-lamellar phases, which affect membrane permeability^18^. This might be of biological relevance, especially when considering disorders, such as NPC1, where Sph, which is a positive regulator of calcium release from the acidic stores, abnormally accumulates in acidic compartments^16, 30^. Other mechanisms have been suggested in order to explain this Sph-induced permeability, including the formation of structural defects at the interfaces between biophysically distinct lipid domains^20^, and the formation of pores^31^. Despite this, the molecular mechanisms underlying this phenomenon are not yet completely understood. In addition, most of the model systems used to address the physico-chemical impact of Sph in the membranes fail to mimic the biological properties of one of the most important subcellular locations of Sph – the lysosome – not only in terms of lipid composition, but especially concerning the pH gradient across the lysosomal membrane. Therefore, in the present work, we developed and characterized a synthetic membrane system - the lysosome-mimicking vesicles (LMVs) - in which Sph encounters two different pH environments: an internal acidic pH (pH 5.0), mimicking the lysosomal lumen, and an external neutral pH (7.4), mimicking the lysosomal outer leaflet, i.e., pH 5.0_in_/7.4_out_ (Fig. 1). The LMVs were then used to address the biological relevant question of how Sph accumulation in the lysosome, as observed in NPC1^16^, changes lysosomal membrane properties, both in dynamic and in thermodynamic equilibrium conditions (Fig. 1). The lipid composition of the vesicles has been manipulated in order to compare the effects of Sph on membrane permeability and biophysical properties under situations that more closely resemble the physiological conditions, i.e., low Chol content^32, 33^, and NPC1-like pathological conditions, i.e., high levels of Chol and SLs^3, 16^. Our results showed that Sph has a more dramatic impact on membrane organization and permeability in NPC1-mimicking conditions, compared to physiological-like situations. Moreover, our data further showed significant differences in the effects caused by Sph in LMVs and standard vesicles (with no pH gradient, Fig. 1), further supporting the notion that adequate synthetic systems should be used to address lipid-lipid interactions in conditions that better mimic the biological context.

## Results

### Rationale

The present study aimed at developing synthetic systems that more closely resembled the lysosome, to more accurately address the impact of lipid changes on the biophysical properties of lysosomal membranes in physiological and pathological (NPC1-like) situations. Therefore, to mimic the lysosomal compartment, we developed a model bilayer – the LMVs (Fig. 1) - where the pH environment is acid for the inner membrane leaflet and neutral for the outer membrane leaflet, i.e., pH 5.0_in_/7.4_out_. In addition, we took advantage of the well-characterized POPC/SM/Chol ternary mixtures^34^ in order to evaluate the effect of Sph on the properties of membranes containing low (physiological-like) or high (NPC1-like) levels of Chol and SLs (Fig. 1 and Supplementary Table S1). Studies were performed to address the effects of Sph when in conditions of thermodynamic equilibrium (i.e., pre-incorporated in the membrane prior to vesicle preparation, Fig. 1a.2,b.2,c.2) and upon dynamic interaction with the membrane (i.e., externally added to pre-formed vesicles, Fig. 1a.3,b.3,c.3). In addition, to further show the importance of developing adequate synthetic systems to address lipid-lipid interactions, comparison with standard vesicles (without pH gradient across the membrane) prepared under neutral, i.e., pH 7.4_in_/7.4_out_, and acidic, pH 5.0_in_/5.0_out_, conditions, was performed (Fig. 1), based both on new as well as available literature data^26^.

### Preparation and characterization of lysosome-like models (LMVs)

**Figure 2:**
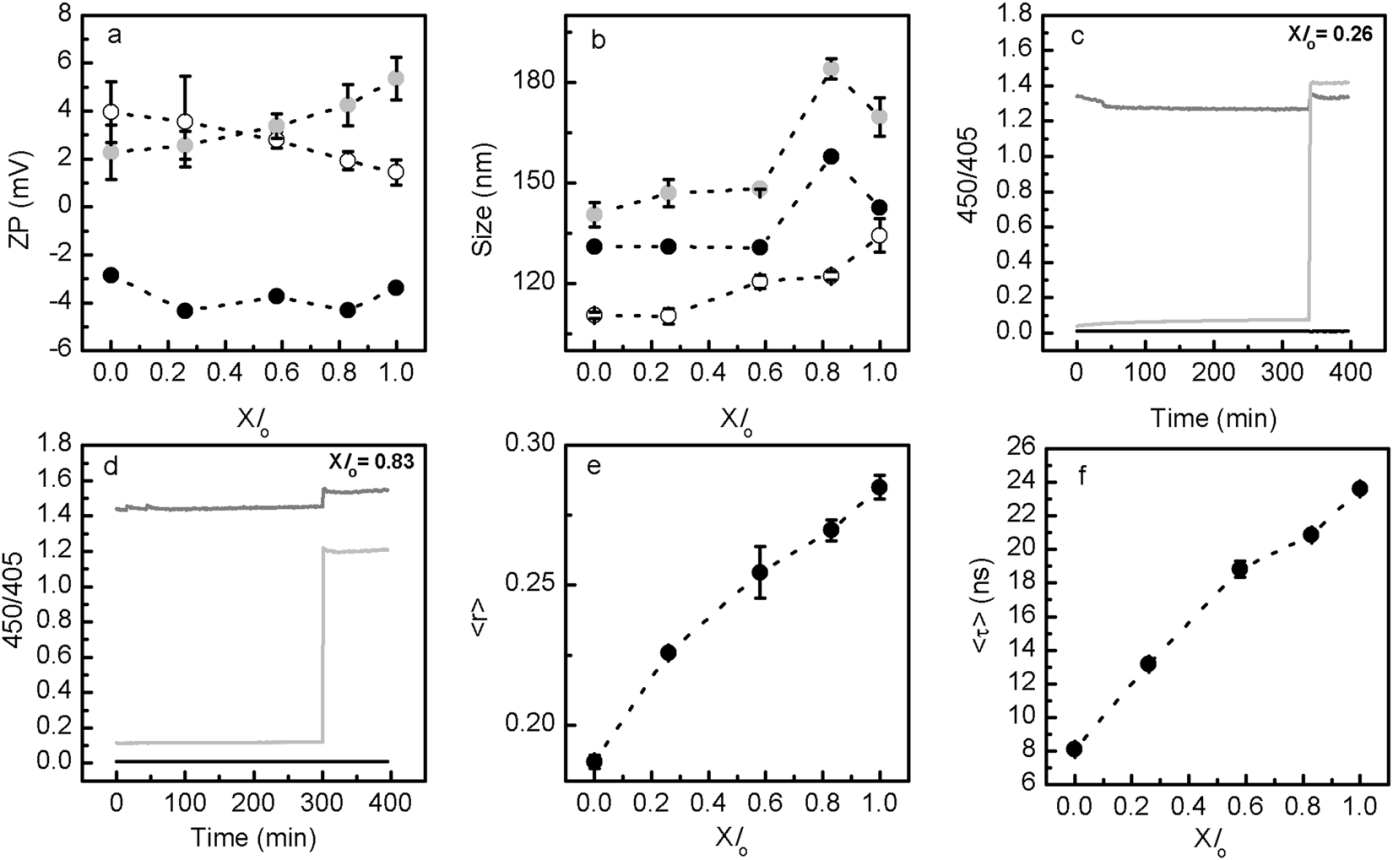
Characterization of POPC/SM/Chol LMVs (pH 5.0_in_/7.4_out_). (**a**) Surface charge and (**b**) size characterization of POPC/SM/Chol vesicles, containing increasing fractions of *l*_o_ phase (see Supplementary Table S1). The open circles in (**a**) and (**b**) represent the samples before chromatography (pH 5.0_in_/5.0_out_), the solid symbols represent the same samples after chromatography (pH 5.0_in_/7.4_out_). The light grey symbols represent the external addition of 10 mol% Sph to POPC/SM/Chol LMVs. (**c**) and (**d**) pH gradient stability of LMVs (pH 5.0_in_/7.4_out_) composed by (**c**) X*l*_o_ = 0.26 and (**d**) X*l*_o_ = 0.83 was evaluated overtime through ratiometric measurements of pyranine excited at 450 and 405 nm (450/405). After approximately 300 minutes, triton X100 (0.1% (v/v)) was added to samples. The light grey line represents LMVs (pH 5.0_in_/7.4_out_) (Fig. 1a.1), while the black and grey lines represent control vesicles prepared with pH 5.0_in_/pH 5.0_out_ (Fig. 1c.1) and pH 7.4_in_/pH 7.4_out_ (Fig. 1b.1), respectively. The vesicles contained 0.5 mM encapsulated pyranine. These experiments were repeated at least three independent times and the values are median representative curves of those experiments. Panels (**e**) and (**f**) show the variation in (**e**) steady-state fluorescence anisotropy and (**f**) mean fluorescence lifetime of t-PnA in ternary POPC/SM/Chol LMVs. In (a,b,e,f) the values are the mean ± SD of at least three independent experiments. The lines act merely as guides to the eye.

The preparation of LMVs (pH 5.0_in_/7.4_out_) (Fig. 1a) is complex involving size exclusion chromatography to create a pH gradient across the bilayer. To test whether this experimental set up would compromise the stability of the vesicles altering its size, and to evaluate if the expected changes in surface charge were taking place upon changing the outer membrane pH from an acidic to a neutral environment^26^, analysis of vesicle size and surface charge was performed before and after separating the vesicles through the chromatographic column (Fig. 2) The ζ-potential of the ternary POPC/SM/Chol mixtures before size exclusion chromatography (i.e., when displaying pH 5.0_in_/5.0_out_) was slightly positive, as expected since these lipid mixtures are at pH of 5.0^26^. A change in ζ-potential towards slightly negative values was observed upon creation of the pH gradient across the bilayer (Fig. 2a), reflecting the charge behavior of these mixtures at pH 7.4^26^.

Changes in the surface charge of the LMVs (Fig. 2a) upon creation of the pH gradient were accompanied by only a slight increase in the size of the vesicles (Fig. 2b), showing that LMVs are stable and not prone to aggregation.

To demonstrate the pH gradient stability of the LMVs (pH 5.0_in_/7.4_out_) we took advantage of the fluorescence properties of the pH-sensitive dye pyranine^35^. The pH gradient in POPC/SM/Chol LMVs was stable over several hours, as shown by the constant ratio of the fluorescence intensity of pyranine (Fig. 2c,d).

To characterize the biophysical properties of the LMVs, fluorescence spectroscopy measurements of trans-parinaric acid (t-PnA) were performed. The partition coefficient (*K*_p_) of t-PnA to POPC and POPC/SM/Chol vesicles at pH 5.0_in_/5.0_out_ and pH 7.4_in_/7.4_out_ was determined as described in Methods section and no significant differences were noticed, regarding both lipid composition and pH environment (See Supplementary Table S2), showing that probe interaction with the membrane is independent of these parameters.

The results of the steady-state and time-resolved fluorescence spectroscopy measurements of t-PnA are represented in Fig. 2 (panels (e) and (f)). An increase in the fluorescence anisotropy (Fig. 2e) and mean fluorescence lifetime (Fig. 2f) of t-PnA was observed in LMVs containing higher *l*_o_ phase fraction (X*l*_o_), reflecting an increase in the order of the membrane as the levels of Chol and SM in the mixtures are increased. Moreover, t-PnA anisotropy (Fig. 2e) and mean fluorescence lifetime (Fig. 2f) in LMVs (pH 5.0_in_/7.4_out_), are in general slightly higher compared to the same mixtures where the internal/external pH was either pH 7.4_in_/7.4_out_ or pH 5.0_in_/5.0_out_^26^ (e.g. the fluorescence anisotropy values for ternary mixtures with no *l*_o_ phase are 0.164 at pH 5.0_in_/5.0_out_; 0.179 at pH 7.4_in_/7.4_out_ and 0.187 at pH 5.0_in_/7.4_out_; Supplementary Fig. S1)^26^, showing that the creation of the pH gradient across the membrane changes lipid-lipid interactions. Therefore, the overall membrane order and organization of the mixtures change with possible consequences for the phase boundaries of the POPC/SM/Chol ternary phase diagram^34^.

### Effect of Sph on membrane properties under thermodynamic equilibrium

**Figure 3:**
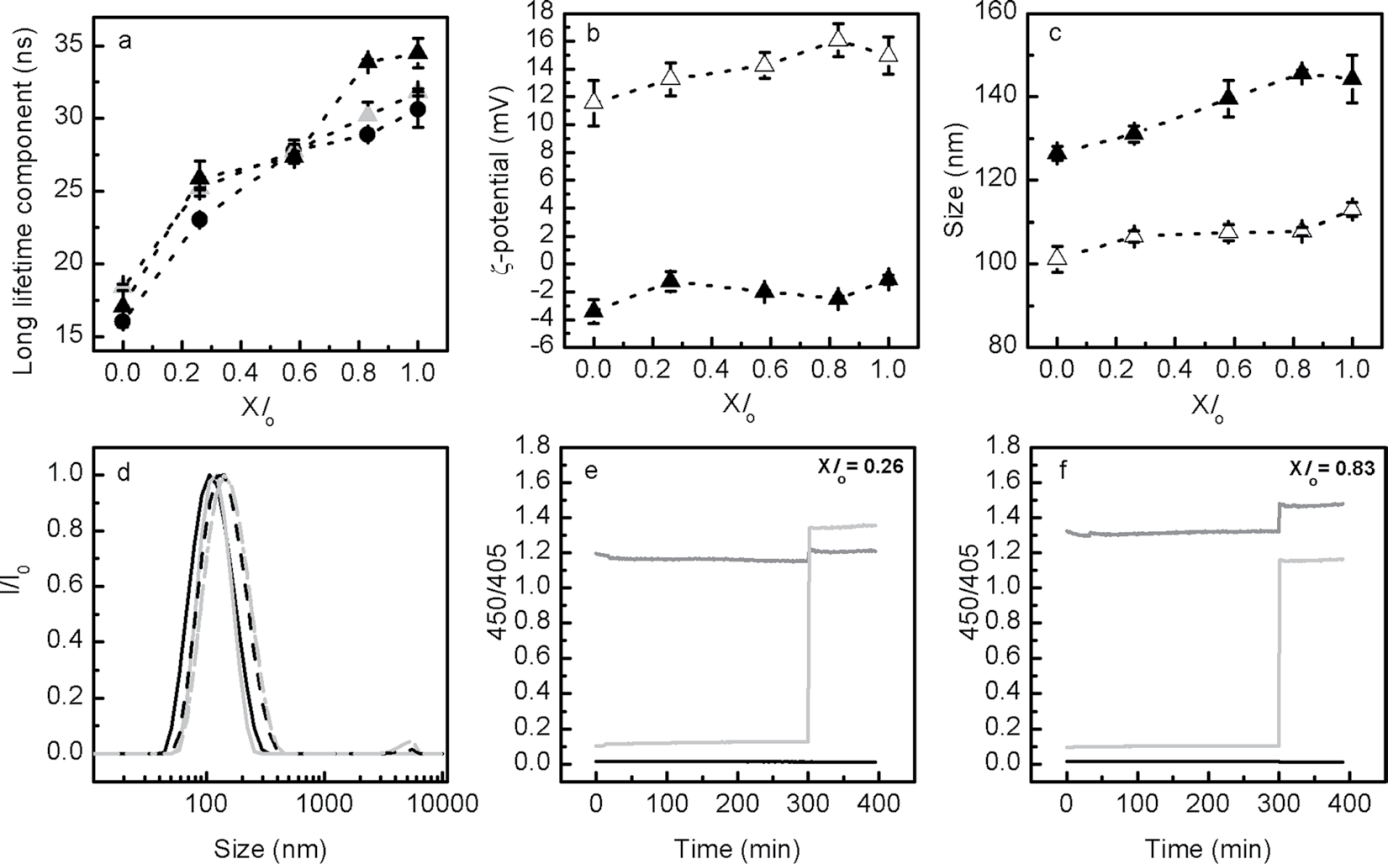
Characterization of POPC/SM/Chol/Sph LMVs (pH 5.0_in_/7.4_out_). Panel (**a**) shows the variation of t-PnA long lifetime component in ternary POPC/SM/Chol LMVs (black circles) and POPC/SM/Chol LMVs containing 5 (grey triangles) and 10 (black triangles) mol% of Sph (pre-incorporated in the vesicles) (Fig. 1a.2). (**b**) Surface charge and (**c**) size characterization of POPC/SM/Chol/Sph (10 mol%, pre-incorporation) vesicles. The open symbols represent the samples before chromatography (pH 5.0_in_/5.0_out_) and the solid symbols represent the same samples after chromatography (pH 5.0_in_/7.4_out_). Panel (**d**) represent the normalized scattered light intensity of POPC/SM/Chol/Sph (10 mol%, pre-incorporation) (Fig. 1a.2) vesicles containing X*l*_o_ = 0.26 (black lines) and X*l*_o_ = 0.83 (light grey lines) as a function of particle size (nm). The measurements were also made before (solid lines) and after (dash lines) size exclusion chromatography. In panels (**a**) to (**c**) data are represented as a function of the *l*_o_ phase fraction (X*l*_o_): the higher the X*l*_o_ the higher the levels of Chol and SM of the mixtures (see methods and Supplementary Table S1 for further details). The values are the mean ± SD of at least three independent experiments. The lines act merely as guides to the eye. In panels (**e**) and (**f**) the pH gradient stability of POPC/SM/Chol/Sph (10 mol% pre-incorporated) LMVs (pH 5.0_in_/7.4_out_) (Fig. 1a.2) composed by (**e**) X*l*_o_ = 0.26 and (**f**) X*l*_o_ = 0.83 was evaluated overtime through ratiometric measurements of pyranine excited at 450 and 405 nm (450/405). After approximately 300 minutes, triton X100 (0.1% (v/v)) was added to the samples. The light grey line represents LMVs (pH 5.0_in_/7.4_out_) (Fig. 1a.2), while the black and grey lines represent control vesicles prepared with pH 5.0_in_/5.0_out_ (Fig. 1c.2) and pH 7.4_in_/7.4_out_ (Fig. 1b.2), respectively. The vesicles contained 0.5 mM encapsulated pyranine. These experiments were repeated at least three independent times and the values are median representative curves of those experiments.

To investigate the effects of Sph in membrane properties under thermodynamic equilibrium conditions, 5 or 10 mol% of Sph was pre-incorporated in the lipid mixtures prior to vesicle preparation (see Fig. 1 and Methods for further details). Pre-incorporation of Sph into the LMVs does not significantly change the fluorescence anisotropy or mean fluorescence lifetime of t-PnA compared to LMVs without Sph (See supplementary Fig. S2). This is in contrast to what has been previously reported for identical mixtures characterized either at pH 7.4_in_/7.4_out_ or pH 5.0_in_/5.0_out_, where a significant increase in membrane order was observed upon pre-incorporation of Sph, particularly in mixtures containing low Chol and SM content^26^. This suggests that under pH conditions mimicking the lysosome, pre-incorporation of Sph at those small molar ratios has no significant effect on the overall membrane order. However, pre-incorporated Sph increases the packing of the ordered phase of the LMVs, as observed by the increase in the long lifetime component of t-PnA fluorescence intensity decay (Fig. 3a). The effect is more pronounced in mixtures containing higher levels of Chol/SM. For these mixtures, the fluorescence lifetime of t-PnA is very long and typical of gel phase^36^. These results differ from our previous observations performed in standard vesicles with no pH gradient (i.e., pH 5.0_in_/5.0_out_ and pH 7.4_in_/7.4_out_), where it was concluded that Sph-ability to form gel domains was higher in mixtures displaying lower *l*_o_ fraction, thus less Chol/SM^26^. This observation further shows that Sph-induced changes on membrane organization are highly dependent on pH.

Surface charge analysis showed that the ζ-potential of the Sph-containing vesicles before size exclusion chromatography, i.e., pH 5.0_in_/5.0_out_, is positive (Fig. 3b) and similar to what we have previously reported^26^. Upon creation of the pH gradient (pH 5.0_in_/7.4_out_) the ζ-potential decreased towards values close to neutrality, which are only slightly lower compared to those previously obtained for Sph pre-incorporated in vesicles prepared at pH 7.4^26^. The creation of the pH gradient also resulted in a shift in the population size towards larger vesicles (Fig. 3c), which was accompanied by a significant polydispersity index (PDI) increase (from <0.1 to >1.5) (data not shown), especially for the mixtures with higher Chol/SM concentration (higher *l*_o_ fraction). This is probably due to increased propensity for vesicle fusion and/or aggregation, as suggested by the scattering intensities (*I*/*I*_0_) recorded in the 1–10 µm range (Fig. 3d). The presence of pre-incorporated Sph in LMVs (Fig. 1a.2) might cause transient membrane instability due to changes in the surface charge and/or redistribution of Sph molecules upon creation of the pH gradient. Nonetheless, pre-incorporation of Sph into LMVs does not affect the pH gradient stability, as shown by the constant ratio of the fluorescence intensity of pyranine (Fig. 3e,f).

### Dynamic interaction of Sph with LMVs and POPC/SM/Chol vesicles with no pH gradient

To evaluate the dynamic interaction of Sph with the membrane and consequent alterations in membrane properties, Sph was externally added to the already formed lipid vesicles (see Fig. 1 and Methods for further details). To address the importance of adequate model systems in the study of lipid-lipid interactions, studies were performed both in LMVs (i.e., pH 5.0_in_/7.4_out_) (Fig. 1a.3) and in standard liposomes display identical internal/external pH (i.e., pH 7.4_in_/7.4_out_ (Fig. 1b.3) and pH 5.0_in_/5.0_out_ (Fig. 1c.3)).

#### Surface charge

Incorporation of Sph in the lipid bilayer upon its addition to the vesicles is expected to cause a change in their surface charge properties, due to the positive nature of Sph. Therefore, from these experiments it is also possible to evaluate the ability of Sph to incorporate the different type of mixtures.

Addition of Sph to LMVs caused a change in vesicle surface charge towards positive values (Fig. 2a). The ζ-potential values obtained under these conditions are in general comparable to those previously obtained at pH 7.4_in_/7.4_out_ for identical mixtures where the same amount of Sph has been initially incorporated in the lipid bilayer prior to liposome preparation^26^. This charge variation reflects the increase in positively charged lipids in the bilayer as a result of Sph incorporation.

**Figure 4:**
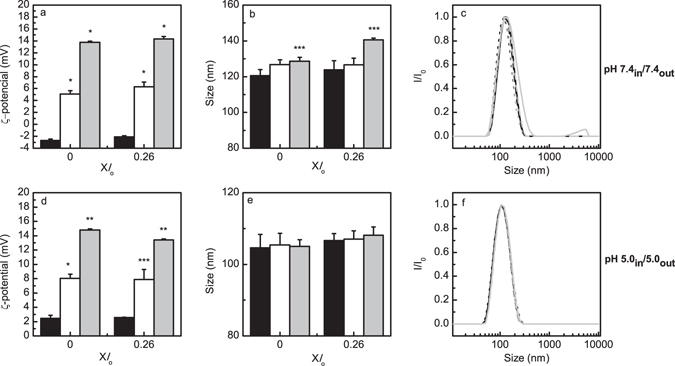
Electrophoretic and dynamic light scattering characterization of POPC/SM/Chol vesicles with no pH gradient. (**a,d**) Surface charge and (**b,c,e,f**) size characterization of POPC/SM/Chol vesicles displaying (upper panels) pH 7.4_in_/7.4_out_ (Fig. 1b.1,b.3) and (lower panels) pH 5.0_in_/5.0_out_ (Fig. 1c.1,c.3). (**a,b,d,e**) The characterization was made before (black) and after the addition of 5 (white) and 10 (light grey) mol% Sph to vesicles containing X*l*_o_ = 0 and X*l*_o_ = 0.26. Panels (**c,f**) represent the normalized scattered light intensity as a function of particle size (nm). The measurements were made before (dot lines) and after the external addition of 5 (dash lines) and 10 (solid lines) mol% Sph to vesicles containing 0 (black lines) and 0.26 (light grey lines) X*l*_o_ phase. The values are the mean (±SD) of at least three independent experiments. *p < 0.001 *versus* 0% Sph; **p < 0.01 *versus* 0% Sph; ***p < 0.05 *versus* 0% Sph.

The effect of adding Sph to standard liposomes prepared under neutral (pH 7.4_in_/7.4_out_, Fig. 4a) and acidic (pH 5.0_in_/5.0_out_, Fig. 4d) conditions, was also evaluated. A Sph-concentration dependent increase in the surface charge of both types of vesicles was observed upon adding Sph, reflecting the incorporation of Sph in the membrane. It should, however, be stressed that, while ζ-potential values obtained at pH 5.0_in_/5.0_out_ (Fig. 4d) are similar to those previously reported for POPC/SM/Chol mixtures containing pre-incorporated Sph^26^, the net surface charge of the vesicles at pH 7.4_in_/7.4_out_ is much higher (Fig. 4a) and comparable to the one measured at pH 5.0_in_/5.0_out_ (Fig. 4d). This difference could be due to a preferential accumulation of Sph in the outer membrane leaflet and/or slow transbilayer movement to the inner membrane leaflet, due to the different protocols used. This would be a valid explanation if the increase of ζ-potential in the presence of Sph was ca. half for pre-incorporation of what was measured for the external addition. However, the ζ-potential increase observed upon external addition is ~3-fold larger for external addition than for pre-incorporation. This can only be explained if Sph is mostly charged when externally added, and not when pre-incorporated. This can be rationalized considering that when the pre-incorporation protocol is used, Sph in the dry lipid - i.e. not ionized - is hydrated together with the other lipids and therefore it is never found in a bulk aqueous environment. In the less polar lipid environment the pKa can shift several units, because the charged form is not being stabilized by the strong ionic character of water^37^.

#### Vesicle size

The external addition of 10 mol% Sph to LMVs led to an increase in vesicle size, especially in mixtures containing higher *l*_o_ content (Fig. 2b). The relatively high PDI (ap. 0.2, data not shown) and the scattering intensities (*I*/*I*_0_) showing a population of particles in the 1–10 µm range (See Supplementary Fig. S3) indicate that these vesicles are prone to fusion and/or aggregation similarly to the observed for LMVs with pre-incorporated Sph (Fig. 3c,d). A similar behavior was observed after the external addition of Sph to POPC/SM/Chol vesicles prepared at pH 7.4_in_/7.4_out_ (Fig. 4b,c) but not for the vesicles prepared at pH 5.0_in_/5.0_out_ (Fig. 4e,f). At pH 5.0 the surface charge of the membranes is slightly positive, becoming more positive upon adding Sph. Therefore, adding Sph will increase repulsion between the liposomes. In case of external pH 7.4, whether the internal pH is 5.0 as in LMVs or 7.4, the initial incorporation of Sph will create areas of the membrane surface that are positively charged, whereas other areas of the membrane still retain the negative surface charge they had prior to Sph addition. This will favor electrostatic attraction between vesicles, and probably facilitate aggregation/fusion events^38^. These events will be limited, occurring only until the distribution of Sph is equilibrated and all the vesicles acquire a similar charge along their surface, and justify the appearance of a population of very large particles in both situations where external pH is 7.4.

#### pH gradient and membrane permeability

**Figure 5:**
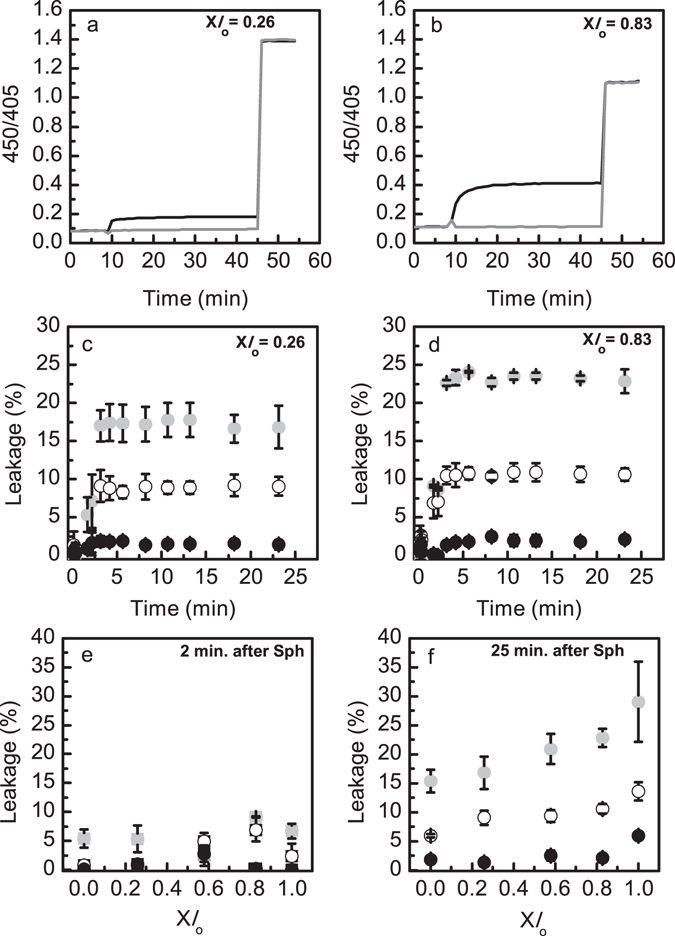
Characterization of POPC/SM/Chol LMVs (pH 5.0_in_/7.4_out_) upon dynamic interaction with Sph. (**a,b**) The pH gradient stability of LMVs (pH 5.0_in_/7.4_out_) composed by (**a**) X*l*_o_ = 0.26 and (**b**) X*l*_o_ = 0.83 was evaluated overtime through ratiometric measurements of pyranine excited at 450 and 405 nm (450/405), after the external addition of 10 mol% Sph (Fig. 1a.3). After approximately 45 minutes, triton X100 (0.1% (v/v)) was added to samples. The light grey lines correspond to LMVs after the addition of ethanol (control samples) and the black lines correspond to LMVs after the external addition of 10 mol% Sph (ap. 10 minutes after LMVs preparation). The vesicles contained 0.5 mM pyranine encapsulated. These experiments were repeated independently, at least three times and these are median representative curves of those experiments. (**c,d**) Sph-induced membrane permeability was evaluated overtime upon adding 5 (white circles) and 10 (light grey circles) mol% of Sph, or ethanol (black circles) to LMVs containing (**c**) X*l*_o_ = 0.26 and (**d**) X*l*_o_ = 0.83. (**e**) and (**f**) show the extent of Sph-induced leakage (**e**) 2 and (**f**) 25 minutes after adding 5 and 10 mol% of Sph, or ethanol (control). The symbols are the same used in panels **c** and **d**. The values are the mean ± SD of at least three independent experiments. The lines act merely as guides to the eye.

The external addition of 10 mol% Sph to pre-formed LMVs (Fig. 5a,b) changed membrane stability and the pH gradient, showing that Sph addition to the vesicles causes membrane permeabilization. This effect was more pronounced for vesicles containing higher *l*_o_ phase fraction (X*l*_o_ = 0.83, Fig. 5b), suggesting that Sph-induced changes in membrane permeability might depend on the initial membrane lipid composition and biophysical properties.

To further address this issue the well-established ANTS/DPX assay was used^18, 20^. Ternary POPC/SM/Chol vesicles were loaded with the fluorescence emitter/quencher pair ANTS/DPX. The release of ANTS/DPX into the aqueous medium results in an increase in the fluorescence intensity of ANTS due to dissociation of the emitter/quencher complex. Therefore, changes in fluorescence intensity are a measure of membrane permeability. The addition of Sph to LMVs, i.e., pH 5.0_in_/7.4_out_, leads to an initial and rapid increase in membrane permeability (up to 2–3 minutes), after which no further changes are observed (Fig. 5c,d). The extent of membrane permeabilization depends both on Sph concentration (Fig. 5c–f) and membrane lipid composition (Fig. 5e,f). Interestingly, and in agreement with the pH gradient stability assays (Fig. 5a,b), the effect of Sph is slightly more pronounced for membranes containing higher content of Chol and SM (Fig. 5f), i.e., in NPC1-like lysosomes, suggesting that accumulation of Sph and Chol in NPC1 lysosomes might lead to higher changes in lysosomal membrane permeability. Control experiments showed that membrane permeability did not change significantly upon addition of ethanol, confirming that increased membrane permeabilization was due to Sph (Fig. 5a–f).

**Figure 6:**
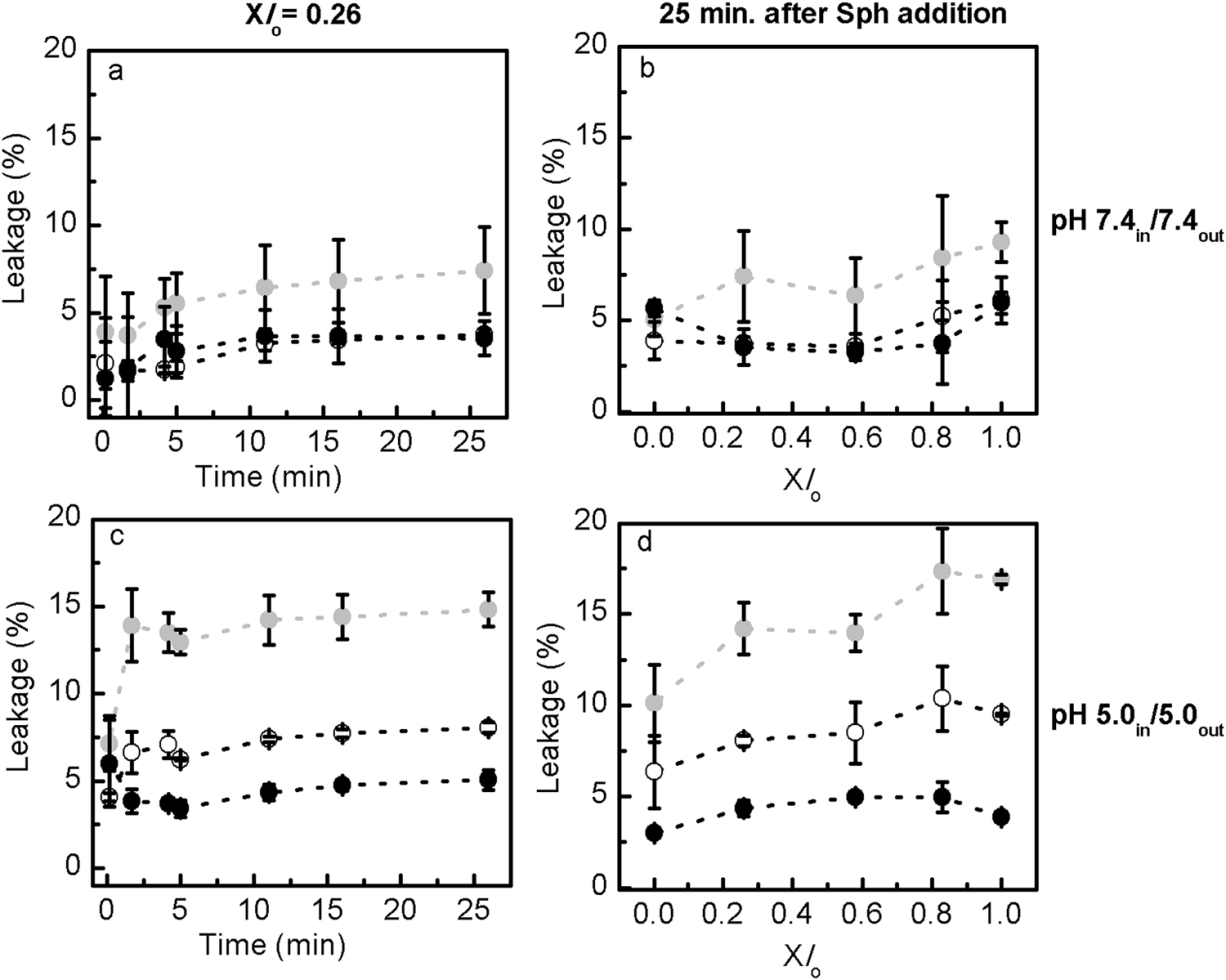
Sph-induced membrane permeability on POPC/SM/Chol vesicles with no pH gradient. (**a,c**) Sph-induced membrane permeability was evaluated upon adding 5 (white circles) and 10 (light grey circles) mol% of Sph, or ethanol (control, black circles) to POPC/SM/Chol vesicles displaying (upper panels) pH 7.4_in_/7.4_out_ (Fig. 1b.3) and (lower panels) pH 5.0_in_/5.0_out_ (Fig. 1c.3) containing X*l*_o_ = 0.26. (**b,d**) Show the extent of Sph-induced leakage 25 minutes after adding 5 (white circles) and 10 (light grey circles) mol% of Sph, or ethanol (control, black circles). The values are the mean ± SD of at least three independent experiments. The lines act merely as guides to the eye.

To address whether the effect of Sph was influenced by the pH environment of the vesicles, studies were also performed in vesicles displaying pH 7.4_in_/7.4_out_ (Fig. 1b.3) and pH 5.0_in_/5.0_out_ (Fig. 1c.3). Figure 6 shows that Sph-induced permeability depends on Sph concentration, membrane lipid composition of the vesicles and pH environment. At pH 7.4 the extent of Sph-induced membrane permeabilization is very low, particularly when 5 mol% Sph are added to the vesicles (Fig. 6a,b). The addition of 10 mol% Sph to the mixtures resulted in a higher leakage, being this effect more pronounced when the *l*_o_ phase is predominant (Fig. 6b), i.e., higher levels of Chol and SM. The extent of leakage induced by Sph under this experimental conditions is comparable to that obtained by Contreras *et al*.^20^, in SM/Chol (80/20) mixtures (pH 7.4) after the addition of the same molar proportions of Sph. Similar results were obtained at acidic pH (Fig. 6c,d), but the extent of leakage is higher at pH 5.0 compared to pH 7.4.

As observed for LMVs (Fig. 5c,d) the effect of Sph addition to vesicles with no gradient is immediate, but at pH 7.4 and pH 5.0 a slight increase in leakage is observed overtime (Fig. 6a,c). However, a similar trend is observed when ethanol is added to the vesicles, suggesting that this small variation is only reflecting the overall permeability properties of the vesicles.

**Figure 7:**
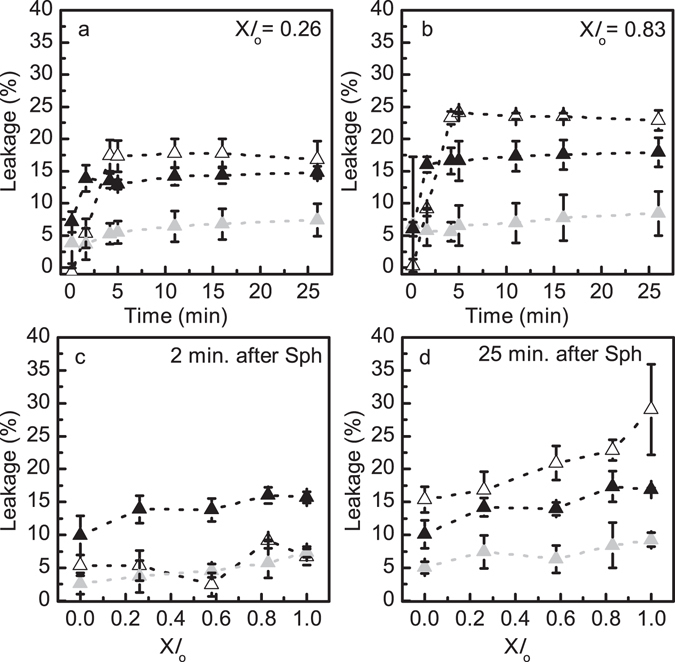
Comparison of Sph-induced membrane permeability in LMVs and POPC/Chol/SM vesicles with no pH gradient. (**a,b**) Sph-induced membrane permeability was evaluated overtime, at pH 5.0_in_/7.4_out_ (white triangles) (Fig. 1a.3), pH 7.4_in_/7.4_out_ (Fig. 1b.3) (light grey triangles) and pH 5.0_in_/5.0_out_ (Fig. 1c.3) (black triangles) after the addition of 10 mol% Sph to POPC/SM/Chol vesicles containing (**a**) X*l*_o_ = 0.26 and (**b**) X*l*_o_ = 0.83. (**c,d**) The extent of Sph-induced leakage was determined in POPC/SM/Chol vesicles containing increasing fractions of *l*_o_ phase (**c**) 2 and (**d**) 25 minutes after Sph addition. The values are the mean ± SD of at least three independent experiments. The lines act merely as guides to the eye.

Further comparison of the effects of Sph on membrane permeability under different pH conditions showed that the extent of leakage was in general lower for vesicles with pH 7.4_in_/7.4_out_, and higher for vesicles mimicking the lysosomal compartment (pH 5.0_in_/7.4_out_) (Fig. 7 and Supplementary Fig. S4). The extent of leakage induced by Sph at pH 5.0_in_/5.0_out_ is intermediate between the two other pH conditions, although closer to the situation mimicking the lysosomal compartment. This suggests that Sph-induced membrane permeabilization is facilitated at acidic conditions. This is further supported by data showing that indeed the initial extent of leakage is higher at pH 5.0_in_/5.0_out_ compared to pH 7.4_in_/7.4_out_ or pH 5.0_in_/7.4_out_, in which Sph first interacts with the membrane exposed to a neutral pH (Fig. 7c and Supplementary Fig. S4c). Indeed, data obtained at pH 7.4_in_/7.4_out_ and pH 5.0_in_/7.4_out_ are very similar (2 minutes after Sph addition; Fig. 7c), which reflects the similar neutral pH conditions sensed by Sph upon its addition to the vesicles prepared at 7.4_in_/7.4_out_ and pH 5.0_in_/7.4_out_. After this initial slower destabilization, a larger increase in the extent of leakage is observed for the LMVs (Fig. 7d and Supplementary Fig. S4d), which is probably due to increased destabilization of the membranes that are exposed to the pH gradient. Therefore, there is a conjugation of different factors that all together contribute to the observed increase in membrane permeabilization upon adding Sph to the LMVs.

#### Sph-induced changes on membrane fluidity and lateral organization

**Figure 8:**
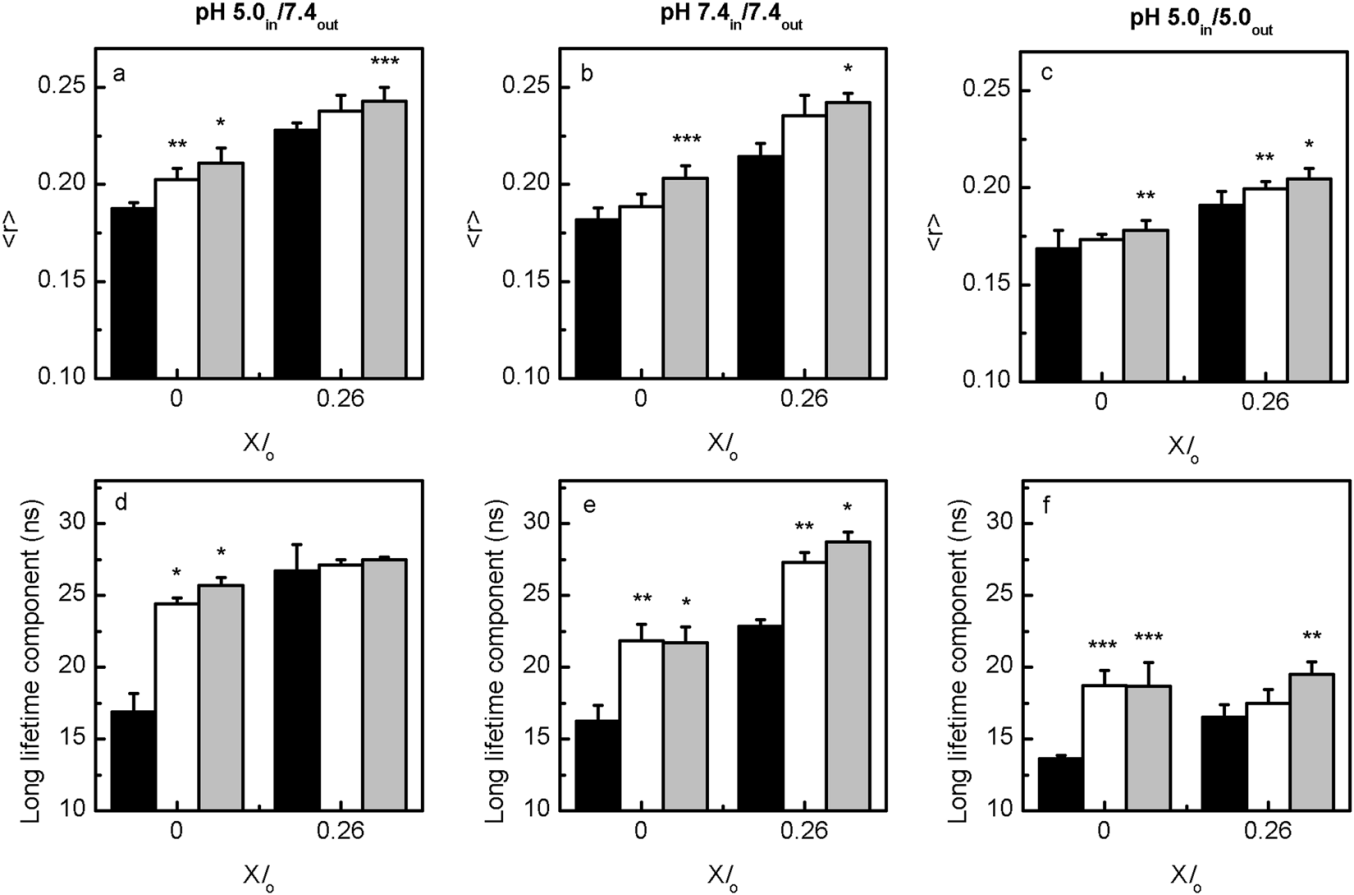
Sph-induced alterations in the biophysical properties of LMVs and POPC/SM/Chol vesicles with no pH gradient. (**a–c**) Steady-state fluorescence anisotropy and (**d–f**) long lifetime component of t-PnA in ternary POPC/SM/Chol vesicles containing X*l*_o_ = 0 and X*l*_o_ = 0.26 before (black) and immediately after addition of 5 (white) and 10 (light grey) mol % of Sph. (left panels, **a,d**) pH 5.0_in_/7.4_out_ (Fig. 1a.3), (middle panels, **b,e**) pH 7.4_in_/7.4_out_ (Fig. 1b.3) and (right panels, **c,f**) pH 5.0_in_/5.0_out_ (Fig. 1c.3). The values are the mean ± SD of at least three independent experiments. *p < 0.001 *versus* 0% Sph; **p < 0.01 *versus* 0% Sph; ***p < 0.05 *versus* 0% Sph.

It has been previously suggested that Sph-induced membrane permeability could be due to Sph-pore formation^31^ or due to increased membrane packing defects as a consequence of lipid phase separation^18, 20^. Therefore, to further characterize the biophysical impact of adding Sph to LMVs, the fluorescence anisotropy and lifetime of t-PnA was measured and compared with the effect of adding Sph to vesicles with no pH gradient (Fig. 8 and Supplementary Fig. S5). The studies were performed for mixtures where a high impact of Sph on membrane properties was observed previously^26^, i.e., mixtures containing lower *l*_o_ phase fraction.

For the LMVs, the t-PnA fluorescence anisotropy was measured 30 minutes (Supplementary Fig. S6), 24 h (data not shown) and immediately (approximately 1 minute) after (Fig. 8a and Supplementary Fig. S6) Sph addition. No significant differences were observed between the different time points, suggesting that the effect of Sph on membrane properties is immediate, likely due to a fast interaction/partition to the membrane.

Addition of Sph to the vesicles caused a Sph-concentration dependent increase in t-PnA fluorescence anisotropy (Fig. 8a–c), irrespective of the pH environment. However, the effect was more pronounced for higher Sph concentrations, at pH 5.0_in_/7.4_out_ (Fig. 8a) and pH 7.4_in_/7.4_out_ (Fig. 8b), reflecting the higher tendency of Sph to increase membrane order at neutral pH^26^. A similar trend of variation was observed upon measuring t-PnA mean fluorescence lifetime (Supplementary Fig. 5a,b), further supporting these conclusions.

To gain further information on the differences in membrane ordering of the mixtures, analysis of the long lifetime component of t-PnA was performed (Fig. 8d–f). Addition of Sph caused a significant increase in t-PnA long lifetime component, particularly at pH 5.0_in_/7.4_out_ (Fig. 8d) and pH 7.4_in_/7.4_out_ (Fig. 8e), in agreement with the observation that Sph-induced membrane ordering is facilitated at neutral pH.

## Discussion

### LMVs: novel lysosome-mimicking systems

To unravel the molecular mechanisms underlying complex cellular events it is often necessary to take advantage of more simple systems that can mimic certain cell features. Artificial membranes are a key example of synthetic systems widely used to address lipid-lipid and lipid-protein interactions, among others^39–41^. The composition of these membranes can be tuned so that specific interactions can be individually studied. The major goal of the present study was to develop a synthetic system with features that more closely resemble one of the major organelles responsible for lipid synthesis and recycling – the lysosome. Such synthetic system constitutes an ideal tool to study how changes in lipid composition derived from both normal and impaired lysosomal lipid metabolism influence membrane structure and biophysical properties. Considering that Sph is a lipid mainly formed by degradation of Cer in the lysosome, and implicated in human disease upon its accumulation in the lysosome^16^, we took advantage of the LMVs (pH 5.0_in_/7.4_out_) (Fig. 1a) to investigate Sph-mediated changes in membrane properties. To this end, the lipid composition of the vesicles was tuned in order to establish a comparison between the effects of Sph in systems mimicking physiological- and NPC1-like conditions.

The LMVs developed in the present study displayed a stable pH gradient across the bilayer, with internal acidic pH (pH 5.0) and external neutral pH (pH 7.4). The surface charge characteristics of the system reflect the neutral pH environment of the outer membrane. The LMVs were stable and not prone to aggregation as verified by vesicle size analysis. The order of the membranes showed differences relative to vesicles with identical lipid composition at only neutral (pH 7.4_in_/7.4_out_) (Fig. 1b) or acidic (pH 5.0_in_/5.0_out_) (Fig. 1c) pH, both in the absence or presence of Sph. This might be due to changes in the lipid-lipid and lipid-solvent interactions^42, 43^. These observations further support that lipid organization, and consequently membrane fluidity, are highly influenced by the surrounding pH environment, and therefore suitable systems to address lipid interactions in a specific subcellular location should be developed to account for organelle-specific features. This is particularly relevant for Sph, where it is already known that this lipid presents different H-bonding states depending on the surrounding environment, shifting from intramolecular to intermolecular between pH 6.7 and 9.9^29^. In addition, it is likely that lipid organization both in the plane of the membrane and across membrane leaflets reflect membrane pH gradient, contributing to the differences in the overall packing of the lipids in the LMVs.

### Application of LMVs to unravel the biophysical impact of abnormal Sph lysosomal accumulation

Our data clearly indicates that the synthetic systems developed herein, with features closer to lysosomes, display distinct properties compared to standard vesicles with no pH gradient. Therefore, those models should be employed to elucidate specific molecular interactions that enable gaining further insight into the biophysical effects derived from Sph lysosomal accumulation under pathological conditions. Hence, we addressed both the dynamic behavior of Sph and its effects under thermodynamic equilibrium conditions, by comparing changes in the ζ-potential, and on the fluorescence properties of the probes. While the ζ-potential relates to the surface charge of the vesicles, and therefore is sensitive to the changes occurring in the outer leaflet of the vesicles, the fluorescent probes are incorporated and distributed within both bilayer leaflets, thus providing biophysical information over the entire bilayer.

The data obtained in the present work pinpoint that the interaction of Sph with the membrane, as well as its effects on membrane order and permeability, strongly depend on the pH environment, inasmuch, as an initial faster permeabilization was observed at acidic pH (pH 5.0_in_/5.0_out_), but the extent of vesicle permeabilization was higher in LMVs (pH 5.0_in_/7.4_out_). Moreover, the results suggest that an increased membrane order, as observed in vesicles presenting an external neutral pH (i.e., LMVs and pH 7.4_in_/7.4_out_ vesicles), can be crucial for an initial protection against membrane destabilization/permeabilization promoted by addition of Sph. Interestingly, data indicate that after the initial minutes upon Sph addition, it seems to exist no direct correlation between Sph-induced changes on membrane order and Sph-induced permeability. Indeed, addition of Sph to the vesicles caused an increase in the overall membrane order, being this effect more pronounced in LMVs and pH 7.4_in_/7.4_out_ vesicles. However, Sph-induced membrane permeabilization was almost negligible in pH 7.4_in_/7.4_out_ vesicles, in contrast to the LMVs that showed the highest extent of membrane permeabilization. Moreover, smaller changes in membrane order were detected in pH 5.0_in_/5.0_out_ vesicles, even though the extent of membrane permeabilization was still significant. Therefore, this suggests that the increase in membrane permeability might not be solely due to structural defects that could be formed at the interface between different phases upon addition of Sph to the vesicles as suggested by other groups^18, 20^. In addition, the hypothesis of permanent pore formation seems to be excluded, since after the initial vesicle destabilization promoted by Sph addition, the pH gradient of the vesicles remained practically unchanged. The extent of Sph-induced membrane permeability might be related not only with membrane order and pH environment, but also to the amount of Sph molecules able to incorporate into the membrane as well as with their distribution between the two membrane leaflets. Indeed, the amphiphilic nature of Sph determines its partition into the membrane.

It is expected that several factors determine the equilibrium between Sph molecules in the aqueous medium and in the membrane. Sph might exist in water both as monomeric species and in the form of micelles, even though the critical micelle concentration (CMC) values of Sph were not yet ascertained (e.g., CMC values ranging from ca. 1 μM to 112 μM have been reported^20, 22, 29, 44^. In addition, Sph partition into the membrane is likely to be affected by the phase properties of the membrane, the electrostatic forces, as well as the properties of the surrounding environment (pH, ion composition, etc). Due to the complexity of this equilibrium it is difficult to correctly determine the extent of Sph membrane partition. In this way, we rationalized our data providing only a qualitative description of the effects of adding Sph to the different vesicles. Thus, we only attempted to compare the effects of externally added Sph to the effect of the same amount of Sph pre-incorporated in vesicles with the same lipid composition. These two scenarios enable comparing the dynamic effects of Sph incorporation on a model of lysosomes, to a situation mimicking a lysosomal membrane enriched in Sph. The former would therefore represent the situation occurring in cells upon interaction of aqueous (free or aggregated) Sph with the lysosomal membrane, which clearly showed that this lipid has the ability to change membrane organization, permeability and surface charge. The latter case would provide the biophysical properties of the membrane with the maximum retention of Sph and with symmetrical distribution of the sphingoid base across the two bilayer leaflets.

Our data shows that irrespective of the pH environment, Sph-induced permeability tends to increase with the extent of *l*_o_ phase present in the vesicles, which seems to be in accordance with the observation made by Contreras *et al*.^20^, that the presence of *l*_o_ regions in the membrane makes it a target for Sph-induced permeabilization. This suggests that Sph interaction with the membrane is facilitated upon increasing the *l*_o_ phase fraction of the vesicles. This could be due to electrostatic forces created between the positively charged Sph and the more electronegative membranes enriched in Chol. Indeed, it has been shown that membranes with higher Chol content are more electronegative due to a lower ability of cations to interact with Chol hydroxyl (-OH) groups^42^. However, the ζ-potential remained constant in LMVs and vesicles with no pH gradient regardless of the Chol content, which may reflect the occurrence of different conformational changes or lipid-lipid interactions^26, 45, 46^. Nonetheless, the external addition of Sph to these vesicles resulted in a larger ζ-potential increase for vesicles with higher Chol content, which suggests a higher incorporation of Sph in the membrane when Chol content is higher. These observations suggest that Sph partition to the membrane is not solely driven by electrostatic forces but it also depends on the lipid composition and membrane biophysical properties.

**Figure 9:**
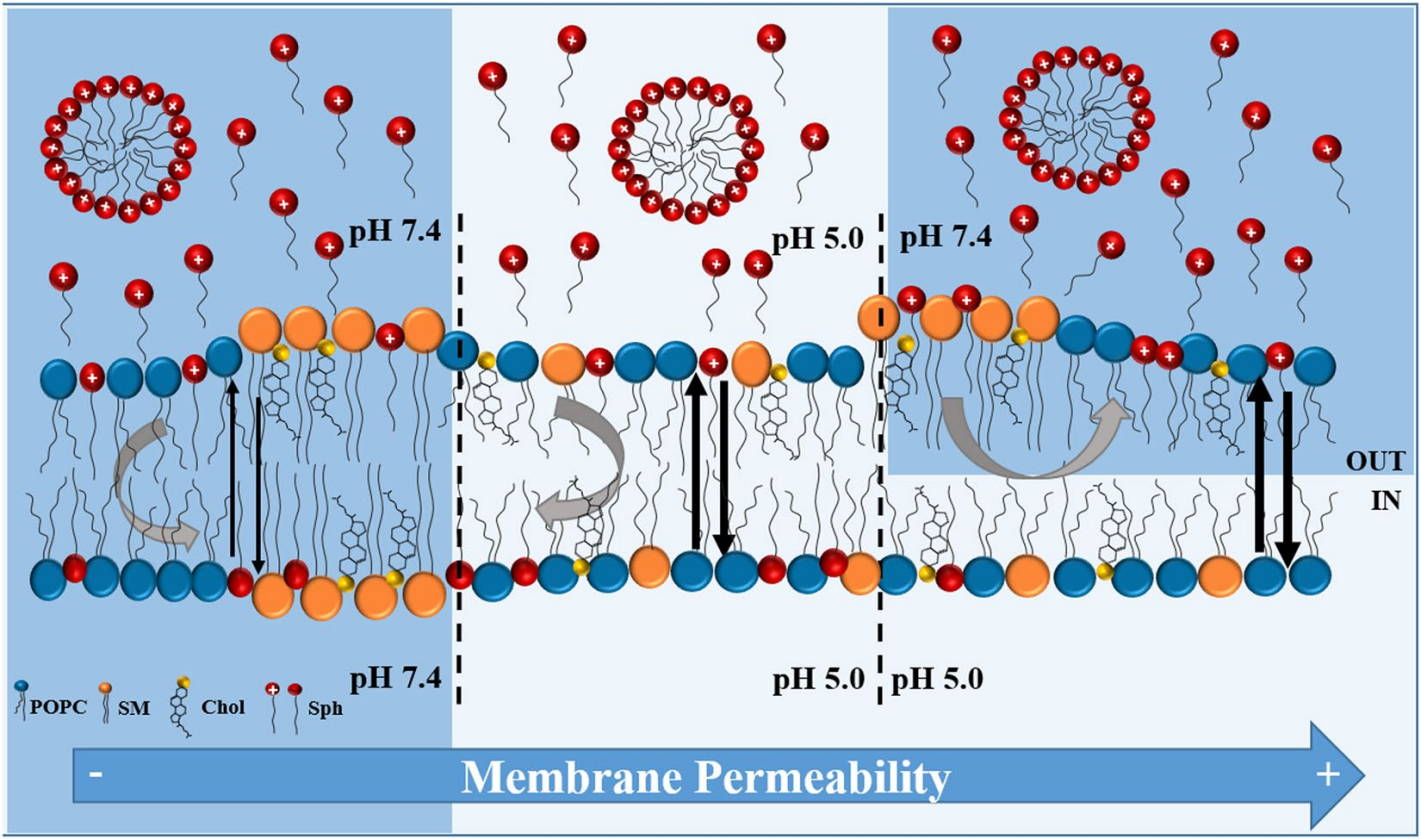
Schematic representation of the effects of external addition of Sph to POPC/SM/Chol vesicles, at different physiological pH conditions. Sph that might exist in water both as monomeric species and in the form of micelles has different patterns of incorporation in the membrane, depending on the pH environment. For pH 7.4_in_/7.4_out_ and pH 5.0_in_/5.0_out_ vesicles the inexistence of a pH gradient across the membrane, favors the creation of a gradient by the positively charged Sph molecules inserted in the outer leafleat, promoting their *flip*-*flop* movement to the inner leaflet, until the gradient is cancelled and the system reaches the equilibrium. In contrast, the pH gradient across the LMVs (pH 5.0_in_/7.4_out_) bilayer decreases the driving force for Sph incorporation. In this case, the distribution of positively charged Sph molecules in the external leaflet of LMVs might generate asymmetries and curvature stress at the membrane. Together with the fact that the inner leaflet of the LMVs is more disordered than the external leaflet, this could justify the increase in membrane permeability. The membrane of the pH 5.0_in_/5.0_out_ vesicles is indeed more disordered compared with the membrane of pH 7.4_in_/7.4_out_ vesicles. Thus, the higher fluidity of the former can favor a faster incorporation of Sph within the membrane and result in an increased rate of membrane permeabilization. The more ordered outer membranes exposed to pH 7.4, would on the contrary contribute to a slower rate of Sph incorporation and prevent membrane destabilization, which is reflect by a lower initial membrane permeabilization. Thin vertical black arrows: low membrane permeability; thicker black arrows: increased membrane permeability. Curved vertical grey arrows: favorable Sph incorporation in the membrane bilayer through *flip*-*flop* movement; horizontal curved arrows: lower driving force for Sph incorporation in the membrane bilayer/higher initial distribution of Sph in the external leaflet. Drawn from left to right: pH 7.4_in_/7.4_out_ vesicles displaying higher membrane order in both leaflets; pH 5.0_in_/pH5.0_out_) displaying lower membrane order in both leaflets; and LMVs (pH 5.0_in_/pH 7.4_out_) displaying higher membrane order in the external leaflet compared to the inner leaflet.

Indeed, POPC/SM/Chol vesicles have a more disordered membrane at acidic pH, which can favor a faster incorporation of Sph within the membrane, leading to the observed higher initial rate of membrane permeabilization. In contrast, the higher membrane order of the vesicles exposed to an outer neutral pH (LMVs and pH 7.4_in_/7.4_out_ vesicles) might decrease the rate of Sph membrane incorporation, resulting in a lower initial membrane destabilization compared to acidic conditions. In vesicles with no pH gradient across the bilayer, the externally added positively charged Sph tends itself to create a gradient across the membrane, followed by *flip*-*flop* movement, until equilibrium is reached. Conversely, in the LMVs, the pH gradient present across the bilayer might decrease the driving force for Sph incorporation. Sph is positively charged and it will not readily move against the charge gradient, that is more positive (<pH) in the interior of the vesicles compared to the external environment (>pH). Thus, the asymmetry between the inner and the outer leaflets created upon the incorporation of Sph in the external monolayer contributes to transient membrane stress and instability (Fig. 9).

Besides changes in membrane permeability, Sph incorporation in the membrane also caused alterations in membrane biophysical properties, leading to an increase in the overall order of the membrane, particularly in the packing of the ordered phase, irrespective of the pH environment. However, Sph was unable to drive gel-fluid phase separation, as previously observed for identical mixtures containing pre-incorporated Sph^26^. This might be due to an effective smaller number of Sph molecules incorporated in the membrane and therefore not enough to induce gel-fluid phase separation. In addition, Sph positive charges can hinder the formation of Sph domains due to the repulsive forces between Sph molecules. According to our surface charge analysis, a significant amount of Sph is located in the outer membrane leaflet, which might create repulsive electrostatic forces that prevent close association between Sph molecules in a gel phase. Sph positive charge might also interfere with the orientation of the head groups and curvature of the neighboring lipids^24^, causing additional defects responsible for increased membrane permeability. As an example the interaction of positively charged Sph with negatively charged phospholipids induce membrane permeabilization through formation of non-lamellar structures^18^.

## Conclusions

The results in the present study highlight the importance of the development of synthetic systems that closely resemble physiological environments. These systems are helpful for better understanding specific molecular interactions that occur in more complex scenarios, namely at the cell level, and even cellular dynamics. Particularly in this study, where systems mimicking physiological and NPC1 like conditions were used to address the interaction of Sph with the membrane, it was possible to detect significant differences as compared to standard vesicles with no pH gradient. Indeed, stronger changes in membrane biophysical properties and permeability were observed upon addition of Sph to vesicles that closely resemble the lysosomal environment (pH 5.0_in_/7.4_out_). This effect was also more pronounced for the vesicles containing higher Chol and SM concentrations, thus mimicking NPC1-membranes. Overall, the results suggest that the abnormal accumulation of lipids in the acidic compartments of diseased cells, might significantly compromise the lysosomal membrane integrity, and consequently affect the normal function of the endolysosomal pathway, and support the further use of LMVs to understand physiological and pathological processes involving the lysosomal membrane.

## Methods

### Materials

Sph, POPC and SM from Egg, Chicken were obtained from Avanti Polar Lipids, Inc. (Alabaster, AL, USA). Chol and TX-100 were obtained from Sigma-Aldrich (St. Louis, MO, USA). *trans*-parinaric acid (*t*-PnA) and pyranine were purchased from Molecular Probes/Invitrogen (Eugene, OR, USA). 8-Aminonaphthalene-1,3,6-Trisulfonic Acid, Disodium Salt (ANTS) and *p*-xylene-bis-pyridinium bromide (DPX) were supplied by Life Technologies (Carlsbad, CA, USA). The organic solvents were obtained from Fluka (St. Louis, MO, USA).

The concentration of the lipid and of the probes stock solutions were determined as previously described^26^.

### Liposome preparation

The lipids were dissolved in chloroform or absolute ethanol (in the case of Sph) and mixed in the required proportions. The following ternary mixtures have been used: 71.6:23.3:5.1; 59.7:26.3:14; 45.1:29.9:25; 34:32.7:33.3 and 25.4:34.8:39.8 (POPC/SM/Chol). These mixtures span the tie line containing the 1:1:1 equimolar mixture of the ternary POPC/SM/Chol phase diagram^34^. For simplicity, data are represented as a function of the *l*_o_ phase fraction of the mixtures: 0; 0.26; 0.58; 0.83 and 1, respectively. To evaluate the effect of Sph on membrane properties, 5 and 10 mol% Sph were also used in the preparation of some samples. Vesicles in thermodynamic equilibrium with pre-incorporated Sph and the dynamic interaction of Sph (external addition) with POPC/SM/Chol vesicles were studied. The solvent was evaporated under a stream of nitrogen and the samples were left under vacuum overnight in order to remove traces of solvent. As suspension medium, Hepes buffer (10 mM Hepes and 150 mM NaCl, pH 7.4) and Citrate Phosphate buffer (0.1 M citric acid and 0.2 M Na_2_HPO_4_, pH 5.0) were used. The lipid suspensions were equilibrated by freeze thaw cycles (T > 50 °C) before large unilamellar vesicles (LUVs) preparation. LUVs (0.2 or 3 mM total lipid concentration, depending on the experiment) were then prepared by standard procedures^47^ e.g. using Nuclepore polycarbonate filters of 0.1 μm pore diameter, at a temperature above the transition temperature of all the individual lipids present in the mixtures (T > 50 °C).

### Preparation of lysosome-mimicking vesicles

To obtain the LMVs, LUVs with a total lipid concentration of 3 mM were prepared with Citrate Phosphate buffer (pH 5.0). To change the external pH environment, LUVs were separated through a Sephadex G-25 gel filtration column (GE Healthcare, Little Chalfont, UK) using Hepes buffer (pH 7.4) as elution buffer. To prevent vesicle burst during gel filtration chromatography, the osmolarity of the buffers was measured using an osmometer (Knauer, Berlin), and when necessary sucrose was added to the elution buffer in order to have identical internal and external buffer osmolarity. Liposomes with pH 5.0 in the internal medium and pH 7.4 in the external medium were recovered mainly in fractions 3 and 4 (1 mL each), as confirmed by absorption, fluorescence and dynamic light scattering measurements (data not shown). The liposomes were then diluted to approximately 0.2 mM lipid concentration.

### Determination of the partition coefficient of t-PnA between aqueous and lipidic phases at different pH conditions

POPC and POPC/SM/Chol (X *l*_o_=﻿ 0.26﻿) vesicles were prepared with different total lipid concentrations (0.001; 0.003; 0.005; 0.01; 0.025; 0.05; 0.1; 0.15; 0.2; 0.25; 0.3; 0.4; 0.5; 1; 2 and 3 mM). The samples were placed in 96 well opaque plates and fluorescence intensity measurements of t-PnA (0.4 µM) were performed at 24 °C, in a microplate reader (Spectramax Gemini EM), using 303 and 404 nm as the excitation and emission wavelengths, respectively. The data analysis software GraFit (Erithacus Software) was used to perform the non linear fitting of equation 1 to the experimental data.

1 $${\rm{\Delta }}I=\frac{(Imax-I0)\times Kp\times [L]}{[W]+Kp\times [L]}$$

In this equation, ∆*I* = *I* − *I*_0_, stands for the difference between the steady-state fluorescence intensity of the probe measured in the presence (*I*) and in the absence of lipid vesicles (*I*_o_). *I*_max_ is the limiting value of *I* measured upon increasing the lipid concentration, [*L*], of the solution, and [*W*] corresponds to the molar concentration of water at 24 °C (approximately 55.5 M)^35^.

### Characterization of membrane biophysical properties

To characterize the biophysical properties of the membranes, steady-state and time resolved fluorescence spectroscopy measurements of t-PnA were performed. Lipid vesicles were incubated with t-PnA (probe to lipid ratio of 1:500) at 24 °C for at least 1 h.

Fluorescence measurements were carried out in a Spex Fluorolog 3-22/Tau 3 spectrofluorometer equipped with double grating monochromators in both excitation and emission light paths from Horiba Jobin Yvon. The t-PnA excitation/emission wavelengths (nm) were 303/404. The fluorescence anisotropy (<r>) was calculated as previously described^48^.

The fluorescence intensity decay measurements were obtained by the single photon counting technique, as previously described^48^. The excitation wavelength was 315 nm (using a NanoLED source, model N-320; Horiba Jobin-Yvon), and the emission was collected at 404 nm. To analyze the experimental decays and obtain the fitting curves, the TRFA software (Scientific Software Technologies Center, Minsk, Belarus) was used. Fluorescence decays were described by a sum of exponentials, where α_i_ is the normalized pre-exponential, and τ_i_ is the lifetime of the decay component *i*. The mean fluorescence lifetime <τ> is given by:

2 $$\langle \tau \rangle =\frac{\sum {\alpha }_{i}{\tau }_{i}^{2}}{\sum {\alpha }_{i}{\tau }_{i}}$$

All measurements were performed in 1.0 cm × 0.4 cm quartz cuvettes, at 24 °C. Constant temperature was maintained using a Braun 852 circulating water bath.

### Leakage studies

LUVs with 3 mM total lipid concentration were prepared (as above described) using Hepes or Citrate Phosphate buffer: (pH 7.4 or pH 5.0, respectively) containing 12.5 mM of ANTS and 45 mM of DPX (leakage buffers)^49^. The non-encapsulated fluorescent probe was separated from the vesicle suspension using a Sephadex G-25 gel filtration column. Buffers with the same osmolarity (adjusted with sucrose as described above) of the leakage buffer but without ANTS/DPX was used as eluent. Liposomes encapsulating ANTS/DPX were recovered mainly in fractions 3 and 4 (1 mL each), as confirmed by absorption, fluorescence and dynamic light scattering measurements (data not shown). Liposome final concentration was determined by lipid phosphorous analysis^50^, for the samples prepared in Hepes buffer. The liposomes were then diluted to approximately 0.2 mM lipid concentration and fluorescence measurements were performed at 24 °C, in 1 cm × 0.4 cm quartz cuvettes under continuous stirring. To evaluate the effect of Sph on membrane permeability, 5 and 10 mol% of Sph, dissolved in a small volume of absolute ethanol (ethanol was kept below to 1% v/v to prevent vesicle destabilization) were added to lipid vesicle suspensions that were in continuous stirring. The same volume of ethanol, without Sph was used as a control. At this ethanol concentration, the permeability change was negligible. Membrane leakage was evaluated by following the increase in the fluorescence intensity of ANTS upon its release from the liposome. To this end, ANTS fluorescence intensity was recorded over time using the same set up described above, using 355/520 nm as the excitation/emission wavelengths. The fluorescence intensity corresponding to full leakage was obtained by adding 0.1% (v/v) of Triton X-100 to the samples at the end of the experiment, i.e., approximately 30 minutes after Sph addition.

The extent of leakage was determined by:

$${\rm{ \% }}\,{\rm{r}}{\rm{e}}{\rm{l}}{\rm{e}}{\rm{a}}{\rm{s}}{\rm{e}}=({{\rm{F}}}_{{\rm{t}}}\,-\,{{\rm{F}}}_{0})/({{\rm{F}}}_{100}\,-\,{{\rm{F}}}_{0})\ast 100$$

where F_t_ is the value of fluorescence intensity at time *t*, F_0_ is the initial fluorescence of the vesicle suspension, and F_100_ is the fluorescence intensity value after the addition of Triton X-100.

### Studies with pyranine

Pyranine is a pH sensitive probe that was used in this work to test the stability of the pH gradient in LMVs. The maximum absorption wavelengths for the acid (protonated) and the base (unprotonated) forms of pyranine are 405 nm and 450 nm, respectively^51^. The fluorescence intensity of pyranine excited at 450 nm is high at pH 7-8 but near background at acidic pH, while the inverse is true for the fluorescence produced by 405 nm excitation^52^. Ratiometric measurements using an excitation ratio of 450/405 nm are for that reason frequently used to provide information about the pH of a determined solution. This is an advantageous method since it not depends on pyranine concentration and is directly related with pyranine ionization degree.

LMVs/LUVs with 3 mM total lipid concentration were prepared using Hepes or Citrate Phosphate buffer (pH 7.4 or pH 5.0, respectively), as above described. These vesicles contained 0.5 mM pyranine encapsulated^35^. The following (POPC/SM/Chol ternary mixtures were used: 59.7:26.3:14 (X*l*_o_ = 26) and 34:32.7:33.3 (X*l*_o_ = 0.83). Liposomes encapsulating pyranine were recovered (after separation through a Sephadex G-25 column) mainly in fractions 3 and 4 (1 mL each). Liposome final concentration was determined by lipid phosphorous analysis^50^, for the samples prepared in Hepes buffer. The liposomes were then diluted to approximately 0.2 mM lipid concentration in 96 well opaque plates and fluorescence measurements were performed at 24 °C, in a microplate reader (Spectramax Gemini EM), using 405 and 450 nm as the excitation wavelengths and 510 nm as emission wavelength. The auto mix option of the microplate reader was selected to mix the samples 5 seconds before the first read and 3 seconds between reads. To evaluate the stability of the pH gradient in LMVs, the fluorescence measurements were performed during *ap*. 5 hours. After this time, triton X100 (0.1% (v/v)) was added to the samples in order to obtain the fluorescence intensity induced by an immediate burst of the vesicles. Some of the wells with LMVs (without the addition of triton X100) were left overnight and measurements were also performed next day, to observe if the pH gradient remained stable. No significant changes were observed (data not shown). Samples with acidic and neutral pH both inside and outside the vesicles were prepared to obtain the fluorescence intensity of pyranine at only acidic and neutral conditions (control samples). The stability of the LMVs was evaluated in the absence and presence of Sph (pre-incorporation of 10 mol% Sph and external addition of 10 mol% Sph). In the studies where Sph is externally added, Sph was dissolved in a small volume of absolute ethanol (ethanol was kept below to 1% v/v to prevent vesicle destabilization) and added to lipid vesicle suspensions (the auto mix option of the microplate reader was selected to mix the samples 5 seconds before the first read and 3 seconds between reads). Control experiments were also performed by adding the same volume of ethanol, without Sph.

### Electrophoretic and Dynamic Light Scattering Measurements

The electrophoretic mobilities were analyzed through M3-PALS technology on a ZetaSizer Nano Z equipment (Malvern Instruments, UK). Samples were placed in clear disposable zeta cells and then in sample chamber maintained at 24 °C. Data analysis was performed using the accompanying software, and the measurements were done in triplicate in each experiment.

Vesicle sizes were determined by performing dynamic light scattering (DLS) analysis on a Zetasizer Nano S equipment (Malvern Instruments, UK). Size measurements were performed using patented non-invasive back scatter (NIBS) technology. Samples were placed in 12 mm square polystyrene cuvettes and then in a chamber maintained at 24 °C. Data analysis was performed using the accompanying software and expressed as Z average size or size distribution by intensity. The polydispersity index (PDI) for each sample was also calculated using the same software. For each sample, the measurements were done in triplicate.

### Statistical analysis

The statistical analysis was performed using Student’s t-test. Mean values were considered significantly different for *p* values below 0.05.

## Electronic supplementary material

Supplementary Information
