# Supplementary material for: Development of lysosome-mimicking vesicles to study the effect of abnormal accumulation of sphingosine on membrane properties
Source: Sci Rep. 2017 Jun 21;7:3949. doi: 10.1038/s41598-017-04125-6 (PMC5479847; doi:10.1038/s41598-017-04125-6)
Supplement: Supplementary file 1 — Supplementary Information [file 41598_2017_4125_MOESM1_ESM.pdf]

Development of lysosome-mimicking vesicles to study the effect of abnormal accumulation of sphingosine on membrane properties

Ana C. Carreira, [§,‡] *Rodrigo F. M. de Almeida,*[‡] *and Liana C. Silva,*[§,†,*]

[§]iMed.ULisboa –Research Institute for Medicines, Faculdade de Farmácia, Universidade de Lisboa,1649-003 Lisboa, Portugal

[‡]Centro de Química e Bioquímica, DQB, Faculdade de Ciências, Universidade de Lisboa, Campo Grande, 1749-016 Lisboa, Portugal

[†] Centro de Química-Física Molecular and Institute of Nanoscience and Nanotechnology, Instituto Superior Técnico, Universidade de Lisboa, Av. Rovisco Pais, 1049-001 Lisboa, Portugal.

Supplementary table S1 – Lipid composition of the vesicles used in this study. Lipid mixtures with five different lipid compositions were prepared. From 1 to 5 the content of both SM and Chol were increased, while the levels of POPC were decreased. This change in membrane lipid composition was made to evaluate the effect of Sph on the properties of membranes containing low (physiological-like) or high (NPC1-like) levels of Chol and SLs. The lipid structures were drawn using Chemdraw software.

**Lipids**	**Lipid Mixtures (mol% of each lipid)**				
	1<br>**Xl_o= 0**	2<br>**Xl_o= 0.26**	3<br>**Xl_o= 0.58**	4<br>**Xl_o= 0.83**	5<br>**Xl_o= 1**
**1-palmitoyl-2-oleoyl-sn-glycero-3-phosphocholine** (POPC)<br>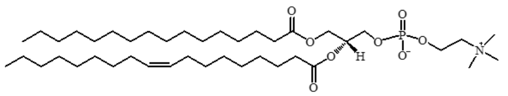	**71.6**	**59.7**	**45.1**	**34.0**	**25.4**
**Sphingomyelin** (SM)<br>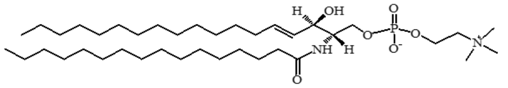	**23.3**	**26.3**	**29.9**	**32.7**	**34.8**
**Cholesterol** (Chol)<br>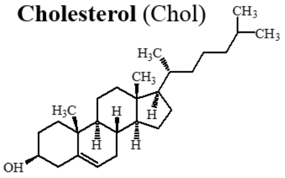	**5.1**	**14.0**	**25.0**	**33.3**	**39.8**
**Sphingosine** (Sph)<br>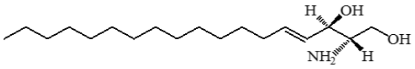	**5.0** or **10.0** mol% Sph<br>(pre-incubation or externall addition to POPC/SM/Chol vesicles)				

Supplementary table S2 – Partition coefficient between aqueous and lipidic phases, K_p, and fluorescence intensity maximum, I_{max}, of t-PnA at different pH conditions.

Vesicles composition	log K_p at pH 5.0	I_{max} (a.u.) at pH 5.0	log K_p at pH 7.4	I_{max} (a.u.) at pH 7.4
POPC	4.74 ± 0.07	482 ± 12	4.30 ± 0.04	509 ± 10
POPC/SM/Chol (26% *l*o)	4.60 ± 0.09	804 ± 22	4.43 ± 0.06	1125 ± 23

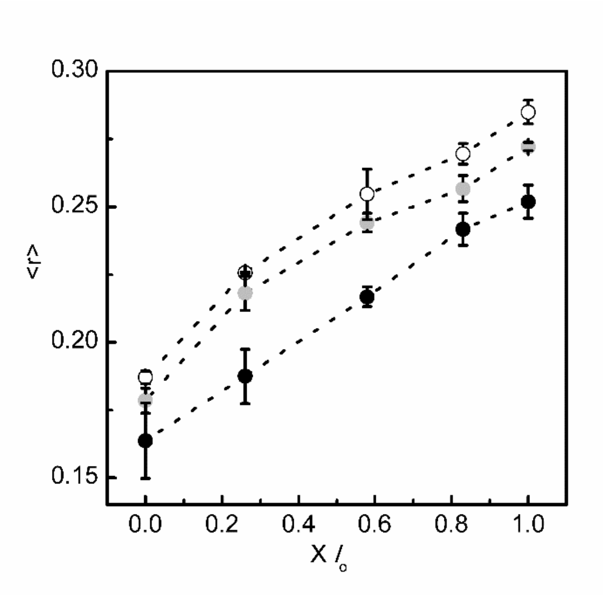

Figure S1 - pH-induced alterations in the biophysical properties of LMVs and POPC/SM/Chol vesicles with no pH gradient. The variation of t-PnA fluorescence anisotropy <r> in ternary POPC/SM/Chol mixtures at pH $5.0_{in}/5.0_{out}$ (black circles), pH $7.4_{in}/7.4_{out}$ (light gray circles) and pH $5.0_{in}/7.4_{out}$ (white circles) is represented as a function of l_o fraction. The values are the mean ± SD of at least three independent experiments.

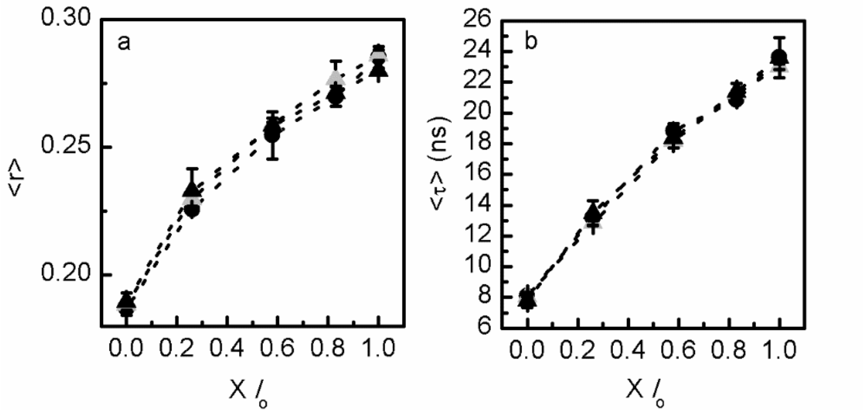

Figure S2 – Biophysical properties of LMVs (pH 5.0$_{in}$ /7.4$_{out}$) in the absence and presence of pre-incorporated Sph. Panels (a,b) show the variation in (a) steady-state fluorescence anisotropy and (b) mean fluorescence lifetime of t-PnA in ternary POPC/SM/Chol mixtures (black) and POPC/SM/Chol mixtures containing 5 (grey triangles) and 10 (black triangles) mol % of pre-incorporated Sph (Fig. 1a.2). Data are represented as a function of the *l*$_o$ phase fraction (X*l*$_o$): the higher the *Xl*$_o$ the higher the levels of Chol and SM of the mixtures (see methods for further details). The values are the mean ± SD of at least three independent experiments. The lines act merely as guides to the eye.

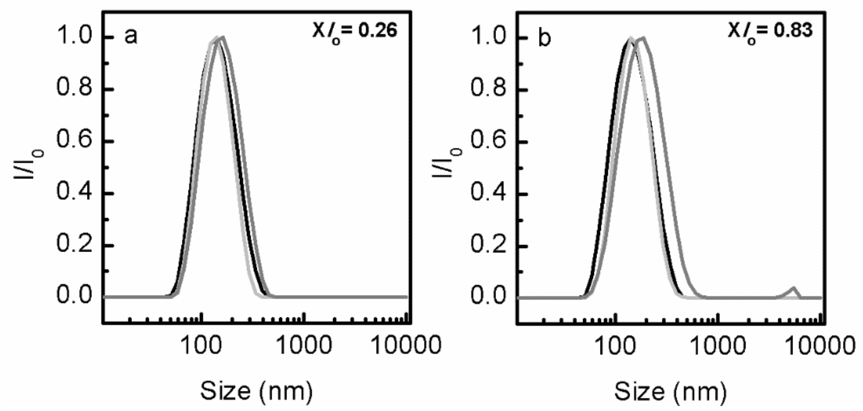

Figure S3 - Dynamic light scattering characterization of POPC/SM/Chol LMVs upon dynamic interaction with Sph. Panels (a,c) represent the normalized scattered light intensity of

POPC/SM/Chol vesicles containing (a) X*l*o = 0.26 and (b) X*l*o = 0.83 as a function of particle size (nm). The measurements were made before (black lines) and after (light grey lines) size exclusion chromatography, and after the addition of 10 mol% Sph to the LMVs (dark grey lines). The values are the mean (± SD) of at least three independent experiments.

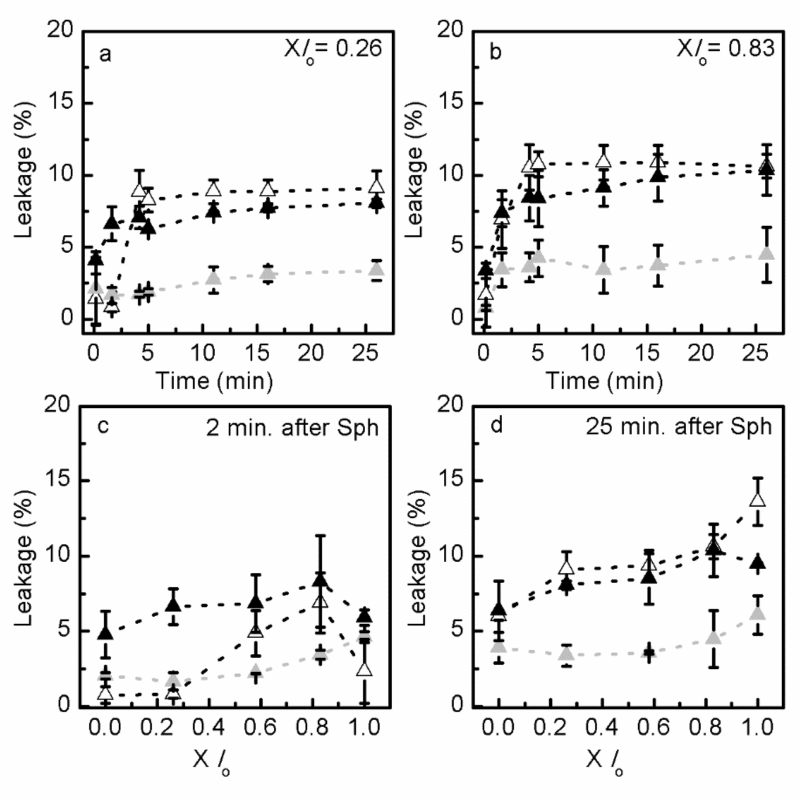

Figure S4 –Sph-induced membrane permeability in LMVs and POPC/SM/Chol vesicles with no pH gradient. (a,b) Sph-induced membrane permeability was evaluated overtime after the addition of 5 mol% of Sph to POPC/SM/Chol vesicles prepared at pH $5.0_{in}/7.4_{out}$ (white triangles), pH $7.4_{in}/7.4_{out}$ (grey triangles) and pH $5.0_{in}/5.0_{out}$ (black triangles). (a) X*l*o = 0.26 and (b) X*l*o = 0.83. (c,d) The extent of Sph-induced leakage was determined in POPC/SM/Chol

vesicles containing increasing fractions of l_o phase (c) 2 and (d) 25 minutes after Sph addition. The values are the mean ± SD of at least three independent experiments.

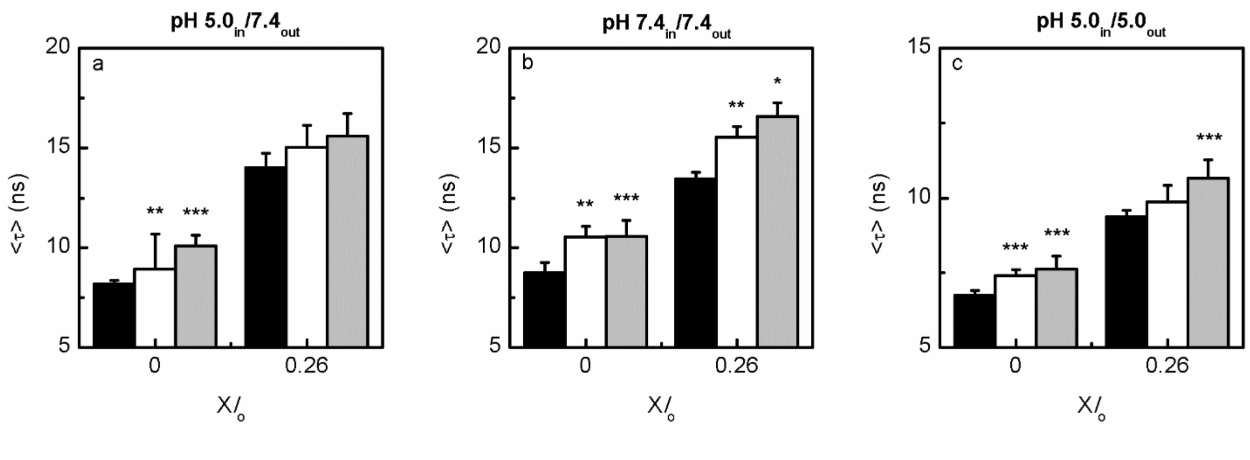

Figure S5 – Sph-induced alterations in the biophysical properties of LMVs and POPC/SM/Chol vesicles with no pH gradient. The variation in the mean fluorescence lifetime of t-PnA before (black) and after external addition of 5 (white) and 10 (light grey) mol% of Sph to POPC/SM/Chol vesicles is represented as a function of l_o fraction. (a) pH $5.0_{in}/7.4_{out}$, (b) pH $7.4_{in}/7.4_{out}$ and (c) pH $5.0_{in}/5.0_{out}$. The values are the mean ± SD of at least three independent experiments. *, $p < 0.001$ *versus* 0% Sph; **, $p < 0.01$ *versus* 0% Sph; ***$p < 0.05$ *versus* 0% Sph.

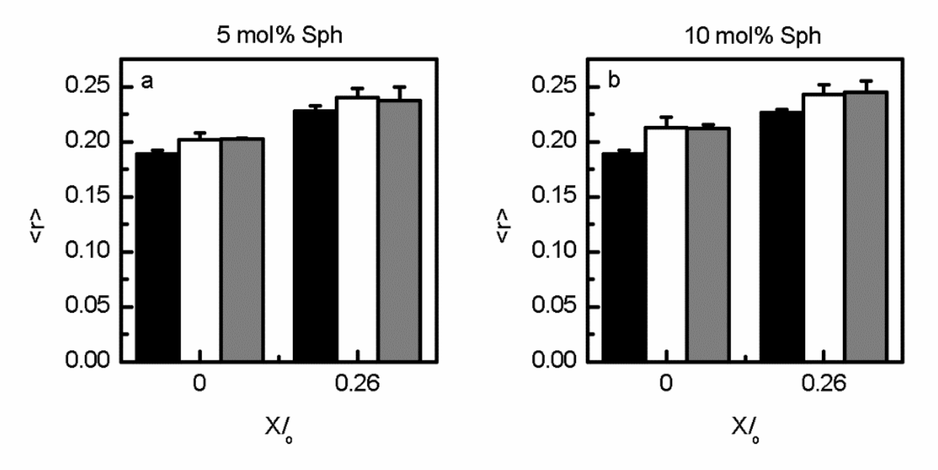

Figure S6 – Sph-induced alterations in the biophysical properties of LMVs. t-PnA fluorescence anisotropy was measured before (black), just after (white) and 30 minutes (grey) after the addition of (a) 5 and (b) 10 mol% Sph to LMVs containing X*l*o = 0 and X*l*o = 0.26. The values are the mean ± SD of at least three independent experiments.
